# Strengthening the Operational Research Capacity of National Tuberculosis Control Programs: Necessity or Luxury?

**DOI:** 10.3390/tropicalmed8070339

**Published:** 2023-06-25

**Authors:** Rony Zachariah, Olga Goncharova, Chynara Kamarli, Timur Bazikov, Sevim Ahmedov, Kudaibergen Osmonaliev, Anthony D. Harries, Hayk Davtyan, Pruthu Thekkur, Gulmira Kalmambetova, Abdullaat Kadyrov

**Affiliations:** 1 United Nations Children Fund, United Nations Development Programme, World Bank, World Health Organization, Special Programme for Research and Training in Tropical Diseases (TDR), 20, Avenue Appia, CH-1211 Geneva, Switzerland; 2 National Center of Phthisiology, Bishkek 720000, Kyrgyzstan; goncharova.ncph@gmail.com (O.G.); abdylat.kadyrov@gmail.com (A.K.); 3 United States Agency for International Development, 171 Prospect Mira, Bishkek 720016, Kyrgyzstan; ckamarli@usaid.gov (C.K.); mohadvisorkg@gmail.com (T.B.); 4 United States Agency for International Development, TB/HIV, Prevention and M&E Team Lead, Bureau for Global Health, TB Division, Washington, DC 20024, USA; sahmedov@usaid.gov; 5 Faculty of Medicine, Ala-Too International University, Bishkek 720000, Kyrgyzstan; kudaibergen.osmonaliev@alatoo.edu.kg; 6 International Union against Tuberculosis and Lung Disease, 2 Rue Jean Lantier, 75001 Paris, France; adharries@theunion.org (A.D.H.); pruthu.tk@theunion.org (P.T.); 7 Department of Clinical Research, Faculty of Infectious and Tropical Diseases, London School of Hygiene and Tropical Medicine, Keppel Street, London WC1E 7HT, UK; 8 Tuberculosis Research and Prevention Center NGO, Yerevan 0014, Armenia; haykdav@gmail.com; 9 Department of Strategic Development and International Cooperation, TB National Reference Laboratory, 90a Akhunbaeva Str., Bishkek 720075, Kyrgyzstan; gulmira.kalmambetova@gmail.com

## 1. The Importance of Embedding Operational Research in Health Systems

*‘How to get research into practice: first get practice into research.’*

John Walley et al. [1]

Many front-line health workers and decision makers in low-and-middle-income countries think that operational research is a luxury that competes for implementation resources [2]. However, it is a necessity, and should be embedded within programs, for three main reasons: First, data that is relevant for improving public health is too often left unused on the shelves or in electronic servers, making disease programs ‘data rich but information poor’. By transforming data into useful information for decision making, operational research can change this paradigm by making countries ‘information rich and action rich’ [3]. Second, there are many tools, innovations and strategies that are generated through clinical trials, but it often takes years to apply them in the real world. Classical examples are insecticide-treated bed nets for preventing vector-borne diseases, which took 18 years from regulatory approval before it was implemented. Similarly, hepatitis B vaccination took 27 years [4]. One of the root causes of such delays is the lack of knowledge on ‘how to’ deliver in the real world; operational research is critical in bridging this knowledge gap [2]. Finally, in 2022, a survey conducted by the World Health Organization showed that 92% of all countries were still facing COVID-19 related disruptions in all service delivery platforms [5]. This shows the importance of ‘real-time intelligence’ to steer the health system out of trouble and sustain the capability of health systems to deliver essential services during pandemics. Operational research can provide such intel [6,7].

## 2. Conducting Operational Research: The Challenge of Lack of Capacity 

Despite its importance, the underlying challenge in conducting operational research is that there is little or no capacity in many countries [1,2,8]. In our opinion, this is due to two reasons. First, existing models for capacity building are often divorced from the delivery of health services. Thus, there is little or no synergy between research and the strengthening of health system capacity to deliver services efficiently. A way to bridge this gap is to try to marry the two—research implementation with research capacity building—so that they happen simultaneously. Second, research is often carried out in isolation and does not involve networks and partnerships, which is key to optimizing resources [9]. Ideally, operational research should engage a range of stakeholders, such as healthcare workers, decision makers, development partners, researchers, and community members, working together to address barriers to healthcare delivery and access. The end goal is the delivery of more effective, efficient, safe, and equitable care that enhances program performance, strengthens health systems, and improves public health.

## 3. The ‘SORT IT’ Solution

The Structured Operational Research and Training IniTiative (SORT IT) is designed to bridge the gaps in conducting embedded research and the lack of capacity. Its purpose is to build capacity to generate and utilize evidence for informed decision making to improve public health [10]. Its design ensures that research focuses on identified constraints in the health system, with the research being led by individuals embedded therein [10]. SORT IT is a global partnership that is coordinated by the UNICEF, UNDP, World Bank, and WHO Special Programme for research and training in tropical diseases (TDR) [10]. The target audience is health workers and decision makers at all levels of the health system. Unique aspects of the SORT IT model are that implementers are in the driving seat and trainees are provided with hands-on mentorship throughout the journey from the stage of generating research questions, to manuscript writing, to effective research communication for impact [11]. 

Since 2012, SORT IT has been scaled up to 94 countries; the initiative now includes 70 partner institutions, has involved 25 public health domains, and about 70% of completed research has been reported to influence policy and/or practice [10,12,13]. 

## 4. SORT IT in the National Tuberculosis Program of the Kyrgyz Republic 

In order to achieve the targets of the End TB strategy, all countries, including the Kyrgyz Republic, need to (a) ensure that TB diagnostic and treatment services reach at least 90% of key populations, and (b) achieve a minimum of 90% treatment success in such populations [14]. Operational research plays a critical role in identifying challenges, proposing solutions, and measuring progress towards achieving these targets. Towards this end, a SORT IT was launched in 2022 in the Kyrgyz Republic focused on health workers within the National Tuberculosis Programme (NTP). This initiative aims to (a) generate evidence on successes and challenges in TB management for key populations, and (b) inform national and regional strategies and policies that pave the way for strengthening health systems and improving patient-centered care. 

**Figure 1:**
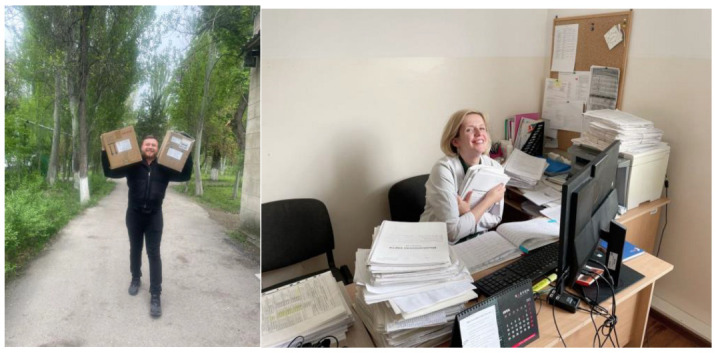
(**Left**) Collection of routinely captured paper-based data from peripheral health facilities and digitization into electronic formats in the central office (**Right**) for operational research, November–May 2023, Kyrgyz Republic (photo credit: Olga Goncharova).

By May 2023, nine policy-relevant research projects stemming from the use of routinely captured health system data (Figure 1) were completed and, are included in this Special Issue of *Tropical Medicine and Infectious Diseases*. Each project was implemented through teams of two to three persons, including training-of-trainers from the Eastern Europe and the Central Asia. The *project strategy* involved harnessing and converging the operational research skills and sharing best practices of regional SORT IT alumni and SORT IT networks in the Eastern Europe and the Central Asian regions with the Kyrgyz Republic (‘think regional, act local’). Notably, all research studies were led by local researchers and were of national relevance (‘local research, local solutions, local ownership’). 

## 5. Where Do We Go from Here: The Next Steps?

1. *Institutionalize operational research within the National TB Programme.* A new unit for research and knowledge management has just been established in the NTP. This is an indication of political will to establish a culture of decision making that is guided through research evidence [15,16]. This unit also creates an opportunity for targeted capacity building and regional and global collaboration.
2. *Build a critical mass of trained researchers.* New cycles of SORT IT are needed to build a critical mass of trained researchers in the NTP who will create an institutional culture of using operational research to inform policy and practice. It will also allow scale-up and integration of research in all geographic areas of the country. An embedded train-the-trainers strategy, by bringing on board those trained in previous SORT IT cycles, would build a pool of mentors and have a multiplier effect. Two to three people could be trained for each SORT IT project. The perspective would be to Train, Embed, Retain, and Enable those trained within the health system [10].
3. *Enhance synergies and the role of academia.* To build a synergy between academic institutions and the NTP, each research project could be led by a pair including a member of staff from the NTP and someone from the academic sector. This will merge different skill sets and have a synergistic effect by integrating SORT IT as part of university curricula and enhancing ‘value for money’.
4. *Knowledge management and research communication:* Researchers should be provided with the skills and tools needed for effective and persuasive communication of research findings [17]. Scientific publications per se need to be transformed into elevator pitches, plain language evidence briefs, and illustrative lectures (short 3-minute and longer 10-minute PowerPoint presentations) for use at national and international forums attended by policy makers. All publications and communication tools will be translated into Russian for effective dissemination.

## 6. Conclusions: The Wider Implications of This SORT IT Initiative 

The focus on TB-key populations is a relatively neglected aspect of TB programmatic work [18,19], and the Kyrgyz Republic can become a front-runner in the generation of useful evidence to inform best practices. This SORT IT initiative is embedded within the health system, promotes local and policy-relevant research, and is led by local investigators. It involves a partnership with institutions from 10 countries who work together to support the Kyrgyz Republic. With a new operational research unit now embedded within the NTP, and the potential for increased involvement with local universities, there is a window of opportunity to build a strong evidence base (and hub) for informing national and regional (World Health Organization) policies. Being in line with the Sustainable Development Goal (SDG) 17.18 for improved use of data for decision making, it can elevate the role of operational research to new heights [8,20].

In conclusion, operational research is not a luxury, it is a necessity that should be geared towards generating evidence that informs policy and practice and strengthens health systems. It should eventually become an integral component of TB services everywhere.
